# Early Motor Development and Rehabilitation Outcomes in Apert Syndrome: Gross Motor Function Measures—Case Report

**DOI:** 10.3390/pathophysiology33010023

**Published:** 2026-03-16

**Authors:** Lorena Oreščanin, Zrinka Biloglav, Ivana Škrlec

**Affiliations:** 1 Physio Kids Centre, 10000 Zagreb, Croatia; 2 Faculty of Dental Medicine and Health, Josip Juraj Strossmayer University of Osijek, 31000 Osijek, Croatia; 3 Department of Medical Statistics, Epidemiology and Medical Informatics, School of Public Health Andrija Štampar, 10000 Zagreb, Croatia; 4 School of Medicine, University of Zagreb, 10000 Zagreb, Croatia

**Keywords:** craniosynostoses, gross motor function measure, motor development, neuroplasticity, physical therapy

## Abstract

Introduction: Apert syndrome is a rare genetic disorder characterized by craniofacial anomalies and limb malformations, often accompanied by neurodevelopmental abnormalities that can considerably affect motor development. Aim: The aim of this study was to document the progress in motor development of a girl with Apert syndrome, with an emphasis on assessing functional needs and evaluating the effects of a multidisciplinary rehabilitation approach. Materials and Methods: Motor functions were evaluated using the Gross Motor Function Measure (GMFM-88) at 16 and 24 months of age. Rehabilitation consisted of an intensive physiotherapy program, Dynamic Movement Intervention (DMI), delivered in monthly cycles over eight months. The therapeutic approach focused on developing postural control, transitional positions, and functional mobility while stimulating sensorimotor integration and neuroplasticity. Results: The initial GMFM score was 29.00%, and the final assessment score reached 68.68%, representing a relative improvement of 136.83%. The most considerable progress was observed in sitting, crawling, and kneeling, with initial improvements in standing. Despite the limitations of this study, the results suggest a positive effect of early, intensive, and individualized rehabilitation combined with active family involvement. Conclusions: The outcomes highlight the importance of early assessment, continuous monitoring of motor development, and a multidisciplinary rehabilitation approach in children with Apert syndrome, with the GMFM serving as a valuable tool for evaluating gross motor function.

## 1. Introduction

Apert syndrome (acrocephalosyndactyly type I) is a rare, genetically determined skeletal dysplasia characterized by multiple craniosynostoses, midface hypoplasia and retrusion, and syndactyly of the hands and feet [1,2,3]. It is named after French pediatrician Eugène Charles Apert (7 July 1868–2 February 1940), who published the first detailed clinical description of the condition in 1906 [4]. The terminology became widely accepted in the mid-20th century, especially after a British series of 39 cases defined Apert syndrome as the typical phenotype of acrocephalosyndactyly [5]. Etiologically, the disorder is the result of a genetic mutation in the *FGFR2* gene (fibroblast growth factor receptor 2) that impairs FGF receptor signaling and leads to abnormal differentiation of osteogenic cells during embryogenesis [1,2]. The syndrome’s distinct phenotypic pattern includes premature fusion of cranial sutures, impaired midface development, complex syndactyly, and malformations of other organ systems. The most commonly identified pathogenic variants in exon seven of the *FGFR2* gene are c.755C>G p.(Ser252Trp, S252W, 176943.0010) and c.758C>G p.(Pro253Arg, P253R, 176943.0011), with the S252W variant being slightly more frequent [6]. Most cases arise de novo, and molecular analyses consistently show a paternal origin of the mutations, with advanced paternal age as a significant risk factor [7]. Epidemiological data on Apert syndrome show heterogeneity across reports. Published studies estimate the incidence at approximately 1:80,000 to 1:160,000 live births, depending on population characteristics and study methodology [8,9,10].

The diagnostic process includes a detailed clinical evaluation, along with targeted molecular genetic analysis of the *FGFR2* gene. Preliminary clinical suspicion is usually based on characteristic phenotypic features [11]. Molecular confirmation of the pathogenic variant enables reliable differential diagnosis from clinically related syndromes, such as those associated with *FGFR3* gene mutations, including Crouzon and Pfeiffer syndromes. Genetic confirmation helps in disease progression prediction and multidisciplinary treatment planning and provides information on reproductive risk assessment and genetic counseling options [1,2,12].

The therapeutic approach requires coordinated, multidisciplinary teamwork, including follow-up with neurosurgical and craniofacial reconstructive procedures, orthopedic limb correction, speech therapy, physiotherapy, and ongoing psychological support for the child and family. Early identification of clinical needs and timely implementation of interventions are essential to optimize functional outcomes, prevent secondary complications, and improve long-term quality of life [13]. Systematic, timely interdisciplinary care enables stable health and a near-normal lifespan despite potential developmental and neurological difficulties [7,9].

Given the rarity and clinical heterogeneity of the syndrome, epidemiological and genetic research play a key role in planning and structuring optimal health care, developing new therapeutic and rehabilitation approaches, and raising awareness of de novo genetic disorders. Systematic literature on the motor development and rehabilitation of children with Apert syndrome using quantitative measures is lacking. This case report study is the first to present progress in the motor development of a 24-month-old girl with Apert syndrome, with an emphasis on assessing functional needs and the effects of a multidisciplinary therapeutic approach.

## 2. Patient and Methods

A case report study involving a girl with Apert syndrome was conducted over eight months and focused on an intensive motor rehabilitation program. The study was approved by Physio Kids Zagreb, where the rehabilitation was conducted. The child’s parents provided written informed consent for her participation and for the publication of the data for scientific purposes, with anonymity ensured.

### 2.1. Clinical Features

The girl was born from the third, spontaneously conceived, and properly monitored pregnancy (gestational age 38 + 4 weeks). At delivery, the mother was 32 years old, and the father was 35. Early combined screening indicated a low-risk finding, while polyhydramnios was noted from the 28th week of gestation. The newborn girl was delivered by cesarean section, with a birth weight of 3440 g, a length of 51 cm, and Apgar scores of 7 and 8 at the first and fifth minutes of life, respectively.

Because of multiple congenital malformations, respiratory distress syndrome, and episodes of apnea requiring mechanical ventilation, the girl was immediately transferred to the intensive care unit. Broad thumbs were present on both hands, along with syndactyly of the second to fifth fingers. Hexadactyly was present on the feet, with syndactyly of the third to sixth toes, leading to clinical suspicion and subsequent diagnosis of Apert syndrome. Family history was negative for hereditary disorders.

The phenotype included pronounced craniofacial dysmorphism: a high forehead and brachycephalic skull shape, a sunken and hypoplastic viscerocranium, a depressed nasal bridge, low-set but regular ears, and retruded eyes. A cleft soft palate was also clinically confirmed. Palpation and imaging revealed a partially separated metopic suture and a widely separated sagittal suture along its entire length (approximately 3 cm), while the coronal and occipital sutures were fused. Because of the atelectasis of the left lung, the newborn was intubated. A tracheal cannula was placed on the 14th day of life, after which spontaneous breathing became possible.

Craniosynostosis of the coronal suture and significant narrowing of the bony part of the choanae were confirmed by imaging diagnostics of the brain, including ultrasound, CT, and MRI. Other developmental anomalies of the central nervous system were also present, including agenesis of the septum pellucidum with fusion of the anterior horns of the lateral ventricles, colpocephaly, dysgenesis of the corpus callosum, and partial hypoplasia of the vermis and cerebellum with formation of an enlarged cisterna magna. Cerebral seizures recorded during the neonatal period were treated with levetiracetam.

Genomic DNA was isolated from peripheral blood using a commercial DNA extraction kit, and exon seven was amplified by polymerase chain reaction (PCR). Sanger sequencing, performed on the Applied Biosystems 3500 Genetic Analyzer (Thermo Fisher Scientific, Waltham, MA, USA), confirmed the presence of the heterozygous pathogenic variant c.755C>G p.(Ser252Trp) in the *FGFR2* gene, thereby molecularly confirming the clinically established diagnosis of Apert syndrome.

### 2.2. Clinical Follow-Up and History of Surgical Procedures

At 6.5 months old, the girl underwent the first surgery, during which a coronal craniotomy was performed with the implantation of a distractor. At 14 months, surgeons performed fronto-orbital reconstruction and remodeling. The third and final craniomaxillofacial surgery is planned for 2027 to reconstruct the middle part of her face.

In addition to reconstructive procedures on her head, in May 2025, the girl underwent surgery on her right hand to correct syndactyly of the second and third fingers. In December 2025, an identical procedure was performed on her left hand, followed by the separation of the first finger from the second and the third finger from the fourth and the correction of the thumb position.

### 2.3. Therapeutic Approach and Motor Rehabilitation

At 16 months old, the girl was referred to the Physio Kids center for professional assessment and developmental support to evaluate her functional status and suitability for inclusion in an intensive rehabilitation program. The therapeutic approach was based on Dynamic Movement Intervention (DMI), a specialized method implemented by trained pediatric physiotherapists and occupational therapists to improve gross motor function in children with neurodevelopmental and neurological disorders. The decision to implement the DMI approach was made after informed consideration of available therapeutic options. Based on research and analysis of data on the effectiveness of these approaches, the parents chose to try DMI in private practice. The decision-making process primarily involved parents and licensed physiotherapists, with consultation and exchange of information about therapeutic goals, options, and expected outcomes. The therapy focuses on facilitating automatic postural reactions through structured, functionally oriented movements guided by the therapist. Precise manual guidance promotes awareness of proper body alignment, balance, weight transfer, and transitions between positions. Simultaneously, repeated performance of challenging yet achievable tasks stimulates neuroplastic processes and the acquisition of new motor patterns. DMI was performed at or above the child’s highest demonstrated functional ability to facilitate higher postural and locomotor patterns. During the sessions, the child was positioned in conditions with modified gravity to stimulate automatic postural responses (righting, protective, and segmental responses), with initial manual facilitation by the therapist that was progressively reduced. The intervention was structured and intensive, with frequent task repetition. Each task was repeated five times per series, and performance was quantitatively recorded as the ratio of successful repetitions (e.g., 3/5) and qualitatively assessed using a standardized ordinal scale (0–3). Therapy progression was based on increasing the difficulty of the position and decreasing the level of manual support.

The girl’s motor status was assessed using the Gross Motor Function Measure (GMFM), a standardized observational instrument and “gold standard” developed to evaluate gross motor function in children with cerebral palsy but also validated for use in other neurodevelopmental disorders, including Down syndrome, spinal muscular atrophy, and several other pediatric conditions [14,15]. The GMFM-88 version was used, and includes 88 tasks divided into five domains: (A) lying and rolling, (B) sitting, (C) crawling and kneeling, (D) standing, and (E) walking, running, and jumping. The instrument enables quantitative evaluation of motor achievement and, due to its strong psychometric properties, is used for initial assessment, longitudinal monitoring of progress, and evaluation of the effectiveness of rehabilitation interventions, including in children with rare neurogenetic disorders [14]. For the GMFM-88, scores can be reported as raw or percentage scores and applied to children with various motor impairments [15].

The primary goal of the therapeutic process was to improve postural control by strengthening the trunk muscles, facilitating righting reactions, and stabilizing the body axis as prerequisites for further motor development. Rehabilitation was conducted for ten consecutive days each month, 45 min per day, with intensive cycles from 14 April to 19 December 2025. The approach was based on the principles of intensive and repeated stimulation of motor learning, with outcomes evaluated after each cycle. Therapy sessions, each lasting 45 min per day, took place in a specialized center with parents present. Parents actively participated in the therapy process and were trained to implement targeted activities at home. Because the parents came from another county, their stay during each cycle had to be organized, and they temporarily took unpaid leave to participate continuously in the child’s therapy. Feedback was provided after each session, and at the end of each cycle, further goals and activities for the next cycle were defined. Through implementation, the child’s functional response was continuously monitored, and the therapy was individually adjusted. The key elements of the approach were individualization, monitoring the response to intervention, and partnership with parents. Therapeutic activities focused on developing transitional positions and body control in space, including changes in position, rotational movements, weight transfers, and controlled destabilizations. These activities targeted stimulation of the vestibular system, stabilization of gaze, activation of trunk muscles, and automatic postural reactions, thereby promoting the integration of sensory and motor information.

Parent education represents an integral part of the therapeutic process. Therapeutic procedures and exercises were explained and demonstrated in detail to the parents to ensure safe and correct implementation at home. On days when the child was not in therapy at the center, the parents performed the same exercises daily at home, ensuring continuous and frequent repetition of rehabilitation activities between sessions. This structured, family-oriented rehabilitation approach is particularly relevant for children with Apert syndrome. A multidisciplinary approach was used in rehabilitation, with the surgical team playing a key role by creating structural and functional prerequisites for more effective physical therapy and further progress in motor development. At the same time, parents actively participated in the therapeutic process by regularly performing exercises at home, ensuring continuity and intensity of the intervention.

## 3. Results

### 3.1. Initial Assessment of Gross Motor Functions

The initial assessment of gross motor function was conducted on 11 April 2025, at 16 months of age. The total GMFM score was 29%, with the highest score in the lying and rolling domain (A) and the lowest in the crawling and kneeling domain (C). The girl achieved no scores in the standing (D) and walking, running, and jumping (E) domains. In the targeted sitting (B) and crawling and kneeling (C) domains, the total score was 26.43%.

Functional analysis showed that the girl rotated independently from supine to prone and primarily moved by “soldier” crawling over very short distances, which was associated with generalized hypotonia and reduced cervical muscle control. In the prone position, she briefly maintains support on her forearms, with limited control of head position. Transition to a side-sitting position is possible only with facilitation. Reduced trunk stability causes her to spontaneously assume a long sitting position with knee extension, which she maintains for a short time. Transitions to quadruped and kneeling positions were possible only with assistance. Additionally, limitations in coordinating rotational trunk movements, reduced mobility of the hands and feet, and difficulty with three-dimensional motor tasks were observed.

### 3.2. Second Motor Function Assessment

The second assessment took place on 19 December 2025, eight months after the initial evaluation. The overall GMFM score was 68.68%, whereas the target domains of sitting (B) and crawling and kneeling (C) combined achieved 87.50% (Table 1).

Considerable functional progress was recorded compared to the initial assessment. The girl independently transitions between lying, sitting, and standing positions; maintains a side-sitting position with effective lateral trunk control; and successfully reaches for objects beyond her base of support. Her primary mode of locomotion is now reciprocal crawling, and during play, she maintains a kneeling position without assistance. Progress was also observed in the standing domain, with the girl standing with minimal support, although there is still a lower tone on the left side of her body.

The overall results of the GMFM assessment show a considerable improvement in functional motor activities, with a percentage change of 136.83% over eight months of rehabilitation (Table 1). Based on these outcomes, the therapeutic focus is now directed toward the standing domain (D), where initial functional changes were observed, with further evaluation planned for the next period.

## 4. Discussion

By applying the DMI therapeutic approach, progress in motor development was monitored using the GMFM in 24-month-old girls with Apert syndrome, and a considerable functional improvement of 136.83% was observed during ongoing rehabilitation. These results suggest a positive impact of early, structured, and targeted rehabilitation focused on functional outcomes, particularly in the context of complex neurodevelopmental disorders.

The effectiveness of the rehabilitation process can be partly attributed to neuroplasticity mechanisms, which enable the reorganization of neural networks, adaptation to altered sensorimotor inputs, and adoption of new motor strategies. Early intervention has the greatest potential for neuroplasticity because, in this period, synaptic connections are strongly influenced by experience and repetition, highlighting the importance of early and targeted neurorehabilitation [16,17,18]. In children with Apert syndrome, neurological abnormalities may further restrict motor development, making systematic rehabilitation from an early age essential [19].

Early surgical interventions, primarily craniosynostosis correction and hand surgery, create functional conditions for neurological and functional development and a foundation for effective rehabilitation [19,20,21,22,23]. Surgical correction of syndactyly is associated with reorganization of somatosensory and motor cortical representations, demonstrating the central nervous system’s capacity to adapt to altered peripheral inputs [24]. This neuroplastic potential is further enhanced by rehabilitation interventions that include tactile and proprioceptive stimulation, differentiated motor tasks, and functional activities, which improve sensorimotor integration and functional independence [25].

Given the pronounced phenotypic and genetic heterogeneity of Apert syndrome, there is no universal therapeutic protocol; instead, an individualized, multidisciplinary rehabilitation approach is required [21]. Innovative methods, such as DMI, may stimulate motor and sensory-integrative functions [26]. In this case, the rehabilitation plan was based on current scientific evidence in physiotherapy for children with neurodevelopmental disorders, emphasizing an activity-oriented, participatory approach and the implementation of therapy in a natural home environment with active family involvement [27]. This approach aligns with current rehabilitation models that move beyond an exclusive focus on body structures and functions, emphasizing functional outcomes relevant to daily life [28].

The GMFM results indicate clinically considerable functional progress; however, interpretation should be considered within a broader developmental context. The literature systematically describing motor development in children with Apert syndrome and related craniosynostosis syndromes is extremely limited, making comparisons with expected developmental patterns difficult [29]. Similar positive outcomes have been reported in case studies of children with rare genetic syndromes, including a 9-year-old boy with *MECP2* (Methyl-CpG-Binding Protein 2) gene duplication syndrome, in whom individualized physiotherapy for eight weeks resulted in a GMFM score of 77.62% [30], and a 2-year-old girl with Pfeiffer syndrome type 2, in whom an overall GMFM function of 84.3% was recorded after 18 months of rehabilitation [27]. These findings further support the importance of an individualized therapeutic approach, in accordance with the principle of goal and intervention specificity [31]. Although it may be tempting to compare the motor outcomes of a child with Apert syndrome to data on the functional abilities of adults with the same diagnosis, such a direct comparison is not possible because the GMFM has been validated only for the pediatric population and has no reference values for adults. However, children with Apert syndrome experience delays in developing fine motor skills and finger awareness (finger gnosis), with functional improvement varying after surgical and rehabilitation interventions [32]. Raposo-Amaral et al. reported reduced hand function, as measured by the Michigan Hand Questionnaire, and delayed performance of fine motor tasks compared to controls after surgical intervention [23]. In adults with Apert syndrome, persistent upper extremity functional limitations have been observed, including prolonged task performance times and compensatory patterns, despite long-term surgical and rehabilitation treatment [33].

The continued involvement of parents in the therapeutic process, along with the child’s exposure to varied sensorimotor experiences in different environments, likely contributed to sustained, multidimensional progress in motor development.

There are several limitations of this study. The GMFM measurement properties for children with conditions other than cerebral palsy are supported by low- to very-low-quality evidence. Therefore, GMFM results should be interpreted with caution when evaluating children outside validated diagnostic groups [14]. Additionally, the single-subject research design prevents generalization of the results, as there is no control group to assess the effect of spontaneous motor maturation. The period from initial to final assessment ranged from 16 to 24 months of age, and some proportion of outcomes may be attributable to the natural course of motor development. However, the final level of functional complexity, particularly in postural control, transitional positions, and initial verticalization, suggests that progress cannot be solely attributed to spontaneous development. In this context, the results indicate a synergistic effect of natural motor maturation and systematic, targeted physical rehabilitation focused on postural stability, sensorimotor integration, and functional mobility.

## 5. Conclusions

This intervention study of a girl with Apert syndrome shows that early, targeted, and systematic rehabilitation, combined with timely surgical intervention, can result in clinically considerable improvements in motor development. Longitudinal monitoring of gross motor function using the standardized GMFM instrument showed a percentage change of 136.83% over eight months of rehabilitation. The findings demonstrate the observed development of an individualized, multidisciplinary, family-oriented rehabilitation approach and highlight the importance of early functional assessment and ongoing evaluation of motor development in children with Apert syndrome.

## Figures and Tables

**Table 1:** GMFM scores before and after physical therapy.

Variables	Baseline—The Raw Score	Baseline	After Physical Therapy, the Raw Score	After Physical Therapy	Percentage Change
Total score		29.00%		68.68%	136.83%
(A) Lying and rolling	47	92.16%	51	100.00%	8.51%
(B) Sitting	26	43.33%	55	91.67%	111.56%
(C) Crawling and kneeling	4	9.52%	35	83.33%	775.32%
(D) Standing	0	0	18	46.15%	∞
(E) Walking, running, and jumping	0	0	16	22.22%	∞

## Data Availability

The data presented in this study are available only on request from the corresponding author due to privacy or ethical reasons.

## Abbreviations

The following abbreviations are used in this manuscript:

GMFM	Gross Motor Function Measure
DMI	Dynamic Movement Intervention
*FGFR2*	fibroblast growth factor receptor 2
